# Volumetric changes in edentulous alveolar ridge sites utilizing guided bone regeneration and a custom titanium ridge augmentation matrix (CTRAM): a case series study

**DOI:** 10.1186/s40729-020-00269-9

**Published:** 2020-12-10

**Authors:** Craig E. Hofferber, J. Cameron Beck, Peter C. Liacouras, Jeffrey R. Wessel, Thu P. Getka

**Affiliations:** 1 Naval Postgraduate Dental School, Bethesda, MD USA; 2 Present Address: Naval Medical Center Portsmouth, Portsmouth, VA USA; 3 Present Address: 3rd Dental Battalion/Naval Dental Center Okinawa, Okinawa, Japan; 4 3D Medical Applications Center, Department of Radiology, Walter Reed National Military Medical Center, Bethesda, MD USA; 5 Radiology and Radiological Services, Naval Postgraduate Dental School, Bethesda, MD USA; 6 Uniformed Services University of the Health Sciences, Bethesda, MD USA; 7 Periodontics Department, Naval Postgraduate Dental School, Bethesda, MD USA; 8 Present Address: Cincinnati, USA; 9 Present Address: Bethesda, USA

## Abstract

**Background:**

The purpose of this study was to evaluate the volumetric changes in partially edentulous alveolar ridges augmented with customized titanium ridge augmentation matrices (CTRAM), freeze-dried bone allograft, and a resorbable collagen membrane.

**Methods:**

A pre-surgical cone beam computed tomography (CBCT) scan was obtained for CTRAM design/fabrication and to evaluate pre-surgical ridge dimensions. Ridge augmentation surgery using CTRAM, freeze-dried bone allograft, and a resorbable collagen membrane was performed at each deficient site. Clinical measurements of the area of augmentation were made at the time of CTRAM placement and re-entry, and a 2nd CBCT scan 7 months after graft placement was used for volumetric analysis. Locations of each CTRAM in situ were also compared to their planned positions. Re-entry surgery and implant placement was performed 8 months after CTRAM placement.

**Results:**

Nine subjects were treated with CTRAM and freeze-dried bone allograft. Four out of the nine patients enrolled (44.4%) experienced premature CTRAM exposure during healing, and in two of these cases, CTRAM were removed early. Early exposure did not result in total graft failure in any case. Mean volumetric bone gain was 85.5 ± 30.9% of planned augmentation volume (61.3 ± 33.6% in subjects with premature CTRAM exposure vs. 104.9% for subjects without premature exposure, *p* = 0.03). Mean horizontal augmentation (measured clinically) was 3.02 mm, and vertical augmentation 2.86 mm. Mean surgical positional deviation of CTRAM from the planned location was 1.09 mm.

**Conclusion:**

The use of CTRAM in conjunction with bone graft and a collagen membrane resulted in vertical and horizontal bone gain suitable for implant placement.

## Introduction

It is well-documented in the literature that resorption of the supporting alveolar bone proper occurs after tooth extraction. Alveolar ridge resorption can account for half of the original bucco-lingual dimension within the first year [1]. Ridge preservation procedures using biomaterials and/or membranes can help preserve ridge dimension after extraction of a tooth [2, 3]. Nonetheless, alveolar ridge defects arising from tooth loss, trauma, or resection of intra-oral lesions often require bone augmentation to facilitate dental implant placement. Creating and maintaining space for bone formation is key to successful ridge augmentation [4].

Fixed, rigid structures such as titanium mesh can be used for this purpose.

Commercially available titanium mesh has been successfully utilized for alveolar ridge augmentation with positive results in both the vertical and horizontal dimensions [5, 6]. Despite the excellent space maintenance properties of titanium mesh, limitations do exist [5, 7, 8]. These include additional intraoperative time needed to trim and adapt the mesh to the site, challenges with adapting mesh to defects for ideal bony contours, and sharp edges on the periphery that may predispose to mucosal perforation and early mesh exposure during healing. The rate of premature mesh exposure reported in the literature was reviewed by Ciocca et al. [9] and ranges from 0 to 52.7%.

Ciocca et al. [10] were the first to describe the integration of computer-aided design (CAD) and 3D modeling to fabricate intra-oral customized titanium meshes (matrices) that provide an intimate and precise anatomic fit to the defect without the need for intraoperative adjustments. A pre-operative cone beam computed tomography (CBCT) scan and CAD were utilized to design a custom mesh, which was subsequently fabricated via direct metal laser sintering (DMLS) of titanium alloy (Ti6AlV4). This approach to augmentation overcomes shortcomings associated with commercially available titanium mesh as well as with other ridge augmentation techniques such as ridge splitting (inability to augment vertically) and autogenous block grafts (patient morbidity [11], graft resorption [12, 13]).

Similarly, Jensen et al. [7] used CAD/computer-aided manufacturing (CAM) to fabricate a customized titanium mesh for alveolar ridge augmentation. Although the amount of augmentation achieved was not quantified, an intimate fit of the mesh to the defect was noted. Comparable findings were reported in a case series by Connors et al. [14] where customized titanium ridge augmentation matrices (CTRAM) were used to achieve ideal restoration of deficient alveolar ridges.

In addition to the precise and intimate fit achieved when utilizing customized titanium mesh, several other advantages have been described in the literature. Eliminating the need to trim and adapt the mesh reduces intraoperative time and eradicates sharp edges along the periphery that may predispose the site to mesh exposure during healing. Sumida et al. [8] compared the average surgical time for customized versus commercially available titanium mesh and found a reduction of 36.52 min when using customized mesh. The rate of mucosal perforation was observed to be 7.7% for customized versus 23.1% for stock mesh with significantly fewer fixation screws required to stabilize the customized mesh.

Multiple recent studies have reported favorable vertical and horizontal linear bone gain at sites augmented with customized titanium mesh [9, 15]. A recent study by Cucchi et al. also evaluated augmentation with customized titanium mesh volumetrically in 10 patients, reporting an average of 89% regeneration of defect area [16]. The aims of this prospective pilot study were threefold: (1) to compare pre- and post-treatment CBCT scans to determine the volume of radiographic bone fill attained, (2) to assess surgical accuracy of the placement of the CTRAM compared to the digitally planned location, and (3) to record bone fill clinically by comparing pre- and post-surgical probing measurements at designated points from the surface of the CTRAM to the underlying bone.

## Materials and methods

The study was approved by the Walter Reed National Military Medical Center IRB (protocol #500069) in compliance with all applicable Federal regulations governing the protection of human subjects. Nine subjects (6 males, 3 females), 42 to 66 years of age, who were non-smokers, and had ASA physical status I or II, were enrolled after informed consent to participate in the study was obtained. All subjects were planned for dental implant treatment and required hard tissue augmentation of a deficient alveolar ridge prior to implant placement.

**Fig. 1:**
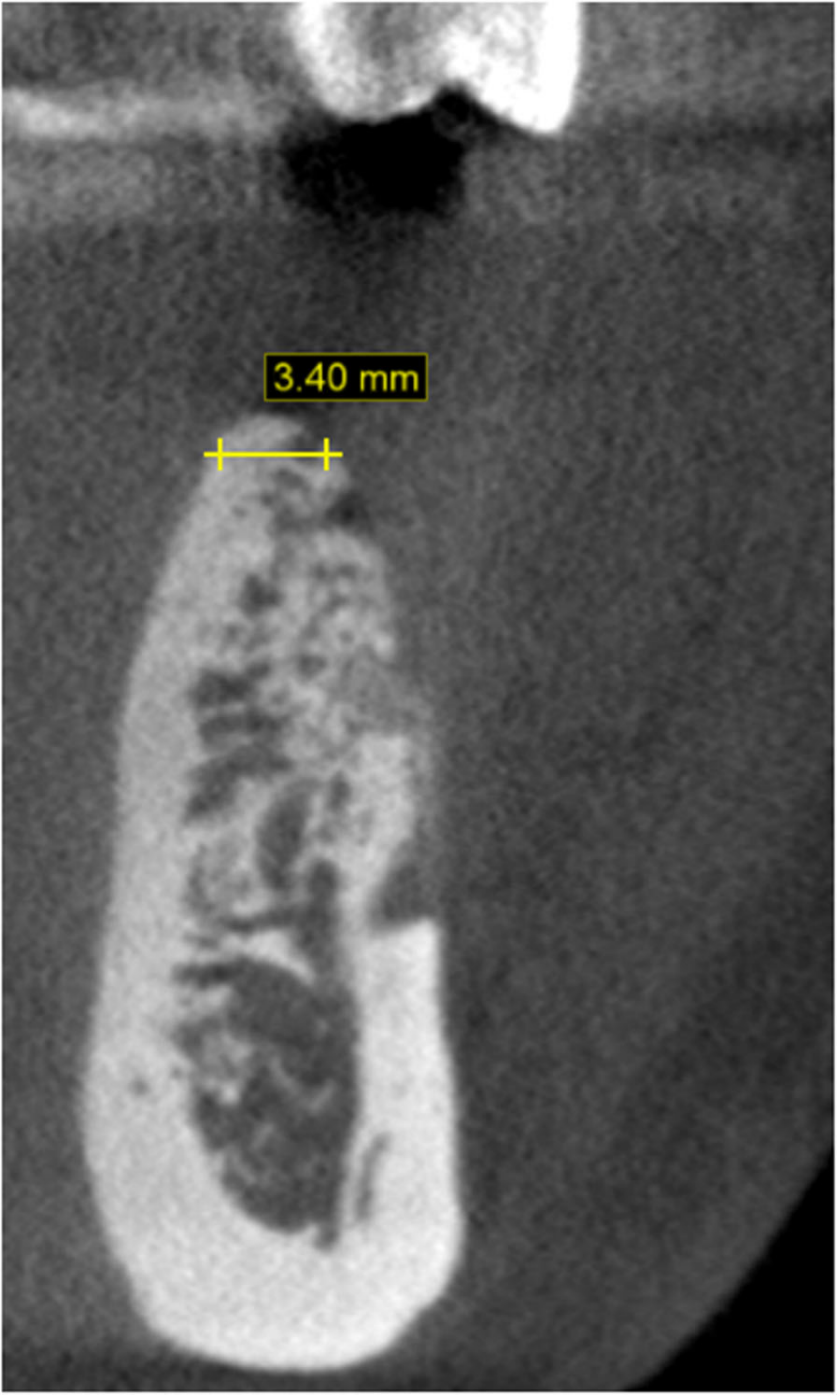
Baseline CBCT demonstrating horizontal ridge deficiency

Baseline CBCT scans (Fig. 1) were obtained and imported into the CAD software (Mimics, Materialise). Thresholding was performed for the osseous tissues, and a stereolithography (STL) file was exported to produce a 3D-printed physical model of the jaw to be augmented.

**Fig. 2:**
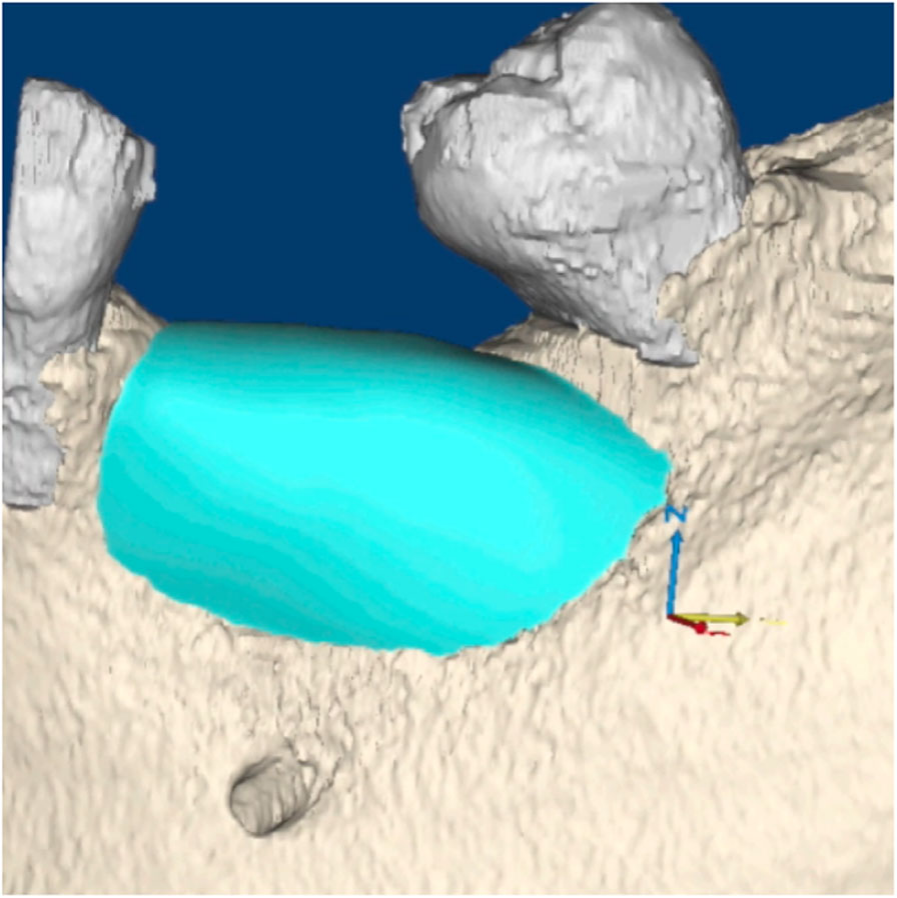
“Ideal” osseous contour digitally added (blue) to the 3D reconstruction of the CBCT scan

**Fig. 3:**
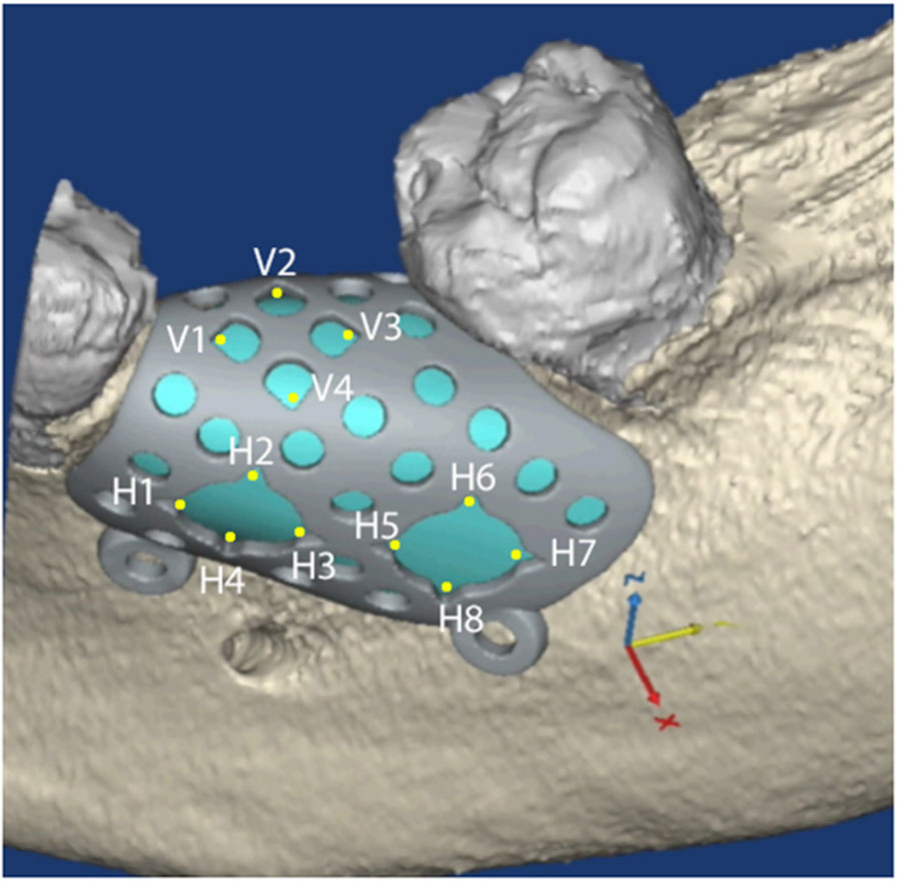
CTRAM designed over the digitally augmented ridge. Notches were designed in bone loading ports at 90° angles were documented as H1–H8 (or H1–H4 if the design included only one loading port). Four cam-shaped nutrient ports were included and documented as V1–V4. These served as measurement locations for pre- and post-graft clinical probing measurements

The STL files of the deficient ridges were then imported into Geomagic Freeform Modeling Plus (3D Systems). A periodontist and engineer worked collaboratively to create an additional digital body file for each case until the precise desired ridge dimension (based on the planned implant positions) was achieved (Fig. 2). The body of the CTRAM was then created over this ideal ridge dimension as a solid surface file with an approximate thickness of 0.5 mm. The CTRAM was then further digitally manipulated to include one or two large bone loading ports to allow placement of graft material, multiple smaller “nutrient” ports to allow for the passage of cells and nutrients from the periosteum to the underlying graft, and two fixation “feet” along the perimeter of the CTRAM for placement of fixation screws. Four notches, at 90° intervals, were designed into the edges of each bone loading port to serve as references for horizontal clinical probing measurements (Fig. 3). Four nutrient ports on the occlusal surface of the CTRAM were also uniquely designed in a cam-shaped fashion to facilitate repeatable vertical probing measurements.

The completed STL file of each CTRAM was exported and digitally processed. The CTRAM was then manufactured on an electron beam melting machine (Arcam) from titanium alloy powder (Ti6AL4V ELI). The resulting product was then cleaned and polished to remove excessive surface roughness and sterilized in an autoclave.

**Fig. 4:**
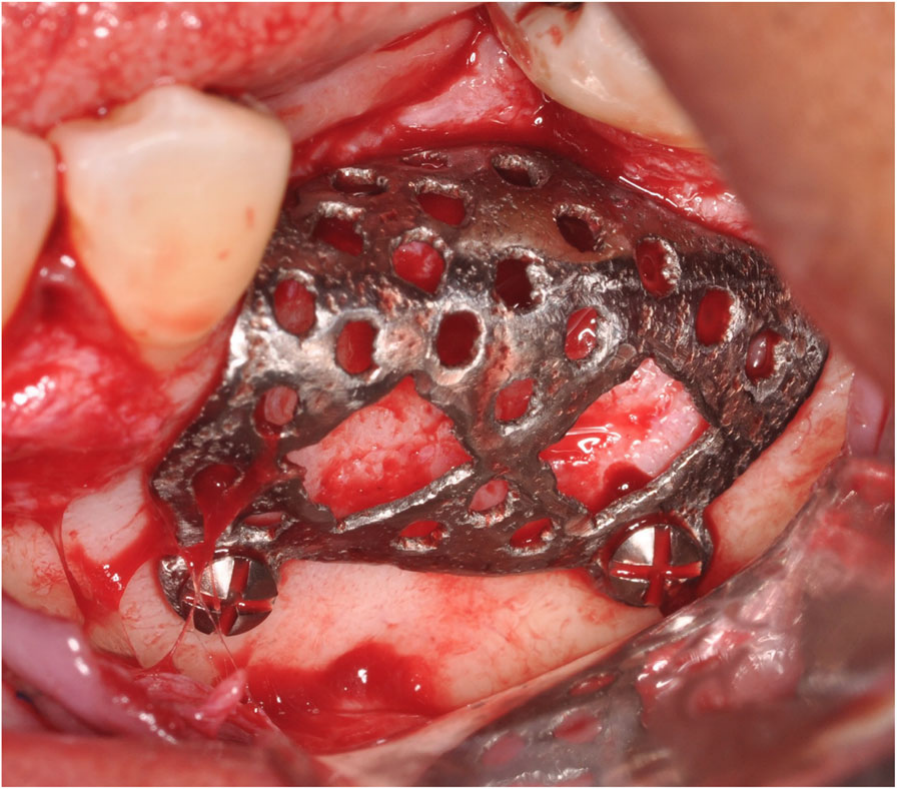
Surgical fixation of CTRAM at site #19 with two screws

Surgeries were performed by first- through third-year periodontics residents. Subjects were administered local anesthesia only, or local anesthesia in conjunction with oral anxiolysis or intravenous moderate sedation. The surgical technique included full-thickness mucoperiosteal flap reflection at the deficient alveolar ridge site followed by CTRAM positioning and assessment for appropriate fit. Intra-marrow perforations were made through the cortical bone in the graft area. The CTRAM were then fixated with either one or two fixation screws as necessary to achieve complete immobilization (Fig. 4).

Following fixation, three designated board-certified periodontists obtained periodontal probe measurements from CTRAM surface to the underlying bone. Measurements were taken to the nearest 0.5 mm with a UNC-15 periodontal probe through the four designated vertical locations and either four or eight horizontal locations on the CTRAM. Freeze-dried bone allograft (FDBA) was then placed through the bone loading ports, and the CTRAM was covered with a resorbable collagen membrane (choice of brand/manufacturer of the membrane was left to each surgical provider). Tension-free primary closure was achieved using monofilament non-resorbable suture material. Subjects were given a standardized post-operative medication regimen consisting of an antibiotic (amoxicillin, or clindamycin if allergic) and analgesics (ibuprofen and hydrocodone/acetaminophen). They were directed to rinse twice daily with a 0.12% chlorhexidine gluconate mouth rinse for 2 weeks. Standard post-operative care was given at weeks 1, 2, 4, and 8.

In the event that soft tissue dehiscence resulted in the exposure of the CTRAM during the post-operative course, surgical providers were given the discretion to treat the areas with local measures (topical application of 0.12% chlorhexidine gluconate) and monitor for signs of adverse healing, or to intervene by removing the CTRAM early.

**Fig. 5:**
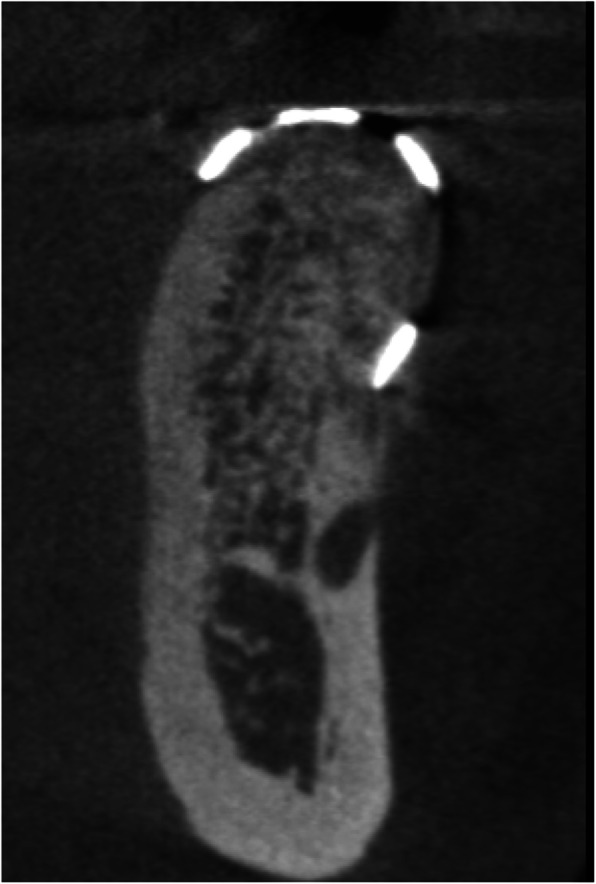
CBCT 7 months post-operatively, suggesting complete bone fill

**Fig. 6:**
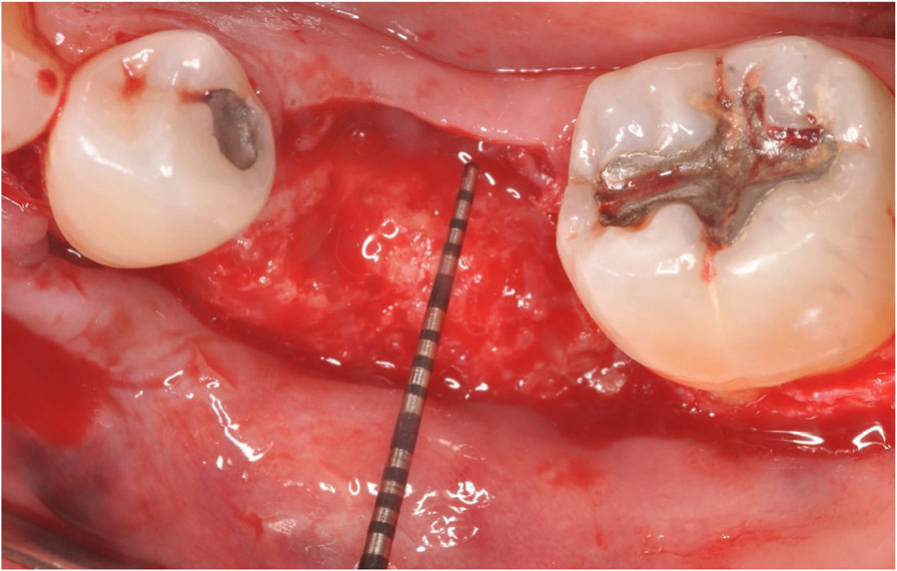
View of ridge after removal of CTRAM. Dimensions of new bone are confirmed to be sufficient for implant placement

A second CBCT scan was taken 7 months post-surgery to assess the degree of bone fill beneath the CTRAM (Fig. 5). At 8 months, a second surgical procedure involving removal of the CTRAM and implant placement was performed (Fig. 6). Surgical steps were completed as previously described to uncover the CTRAM. Clinical probing measurements were repeated at the previously specified vertical and horizontal points. These measurements were taken through the CTRAM to the underlying bone with a UNC-15 probe. CTRAM were then removed and dental implant placement was completed according to the manufacturer’s guidelines.

### Volumetric analysis

**Fig. 7:**
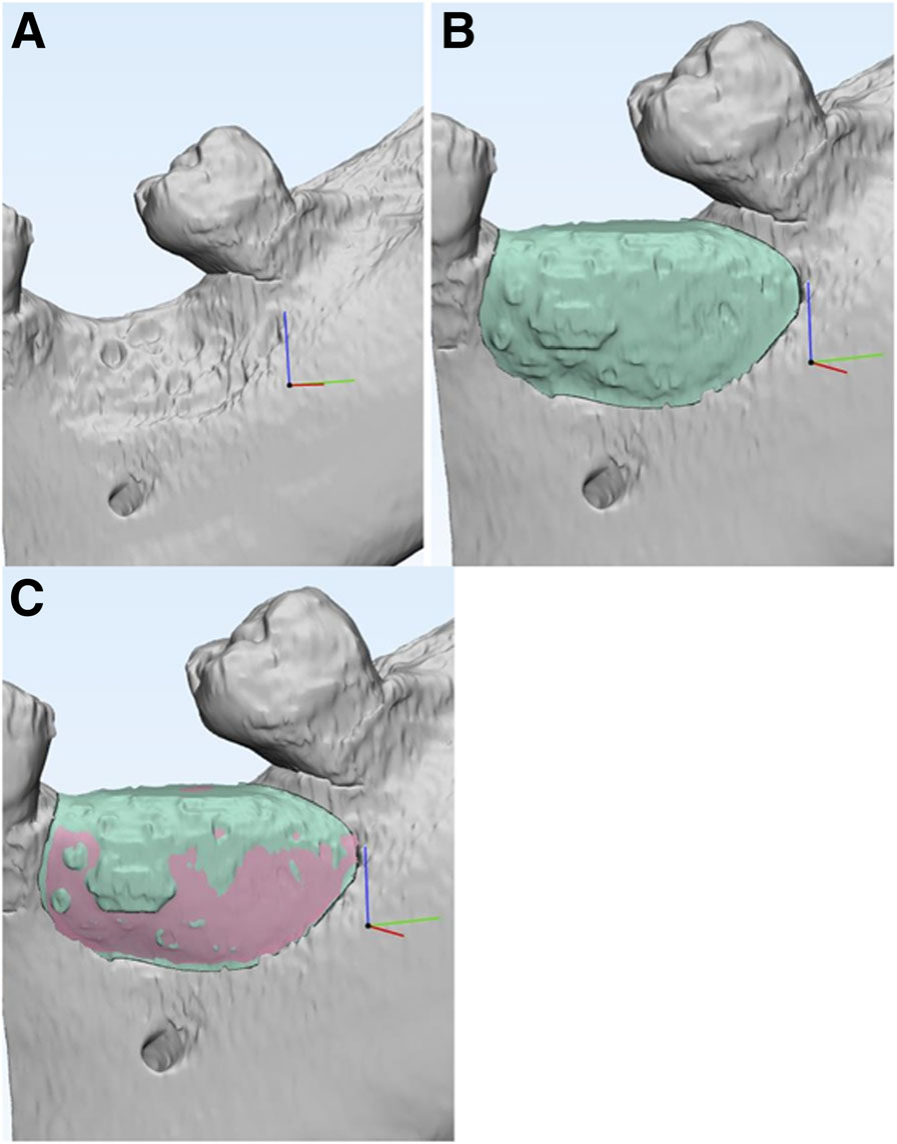
**a** 3D reconstruction of baseline CBCT. **b** Global registration of pre- and post-operative 3D reconstructions; new bone shown in green. **c** Percentage bone fill calculated by comparing planned augmentation volume (pink) with the volume of new bone (green)

3D reconstruction of post-treatment osseous anatomy was completed using the previously outlined CAD methodology based on the 7-month CBCT scan. The CTRAM was segmented separately and maintained as a separate file. Global registration allowed for pre- (Fig. 7a) and post-treatment scans to be overlaid for comparison. Bone fill was measured by comparison of the post-operative osseous dimension (Fig. 7b) with the initial reconstruction (Fig. 7c) using a Boolean subtraction operation. This comparison of the volume of the new bone to the planned volume of augmentation yielded the percentage bone fill. Overall means and standard deviations were calculated, and a Wilcoxon rank-sum test was performed to compare differences in percentage mean bone fill between subjects who experienced soft tissue dehiscence and those who did not.

### Assessment of CTRAM placement

Post-operative reconstruction of the CTRAM, in its surgically fixed position, was performed for each subject. This reconstruction was then transformed globally as a move-along entity, with the osseous registration. To assess the accuracy of CTRAM placement, four points on each subject’s CTRAM were chosen as points for comparison: the mesial and lingual vertical measuring points (V1 and V2) and the most distal and disto-apical horizontal measuring locations (H3 and H4 or H7 and H8 if the CTRAM design contained two bone loading ports). After registering the “planned” and “actual” CTRAM reconstructions and isolating the CTRAM from each, distance measurements were taken from each point to its corresponding point on the other model. Means and standard deviations were calculated on a subject level based on the discrepancies between the four points.

### Clinical assessment of ridge augmentation

Subtraction of the probing measurements obtained at the time of CTRAM removal from the initial probing measurements (at the time of CTRAM fixation) yielded a linear dimension of ridge augmentation. Mean subject level and overall horizontal and vertical augmentation dimensions and standard deviations were obtained by pooling these values.

## Results

No significant complications were noted in any of the subjects. Dental implant placement was completed successfully in all subjects. Of the nine subjects in the study, four experienced premature CTRAM exposure during healing (44.4%). In two of these cases, the CTRAM was removed early at the discretion of the surgical provider, and the fourth was maintained in situ until the prescribed removal time at 8 months.

### Volumetric analysis of ridge augmentation

**Fig. 8:**
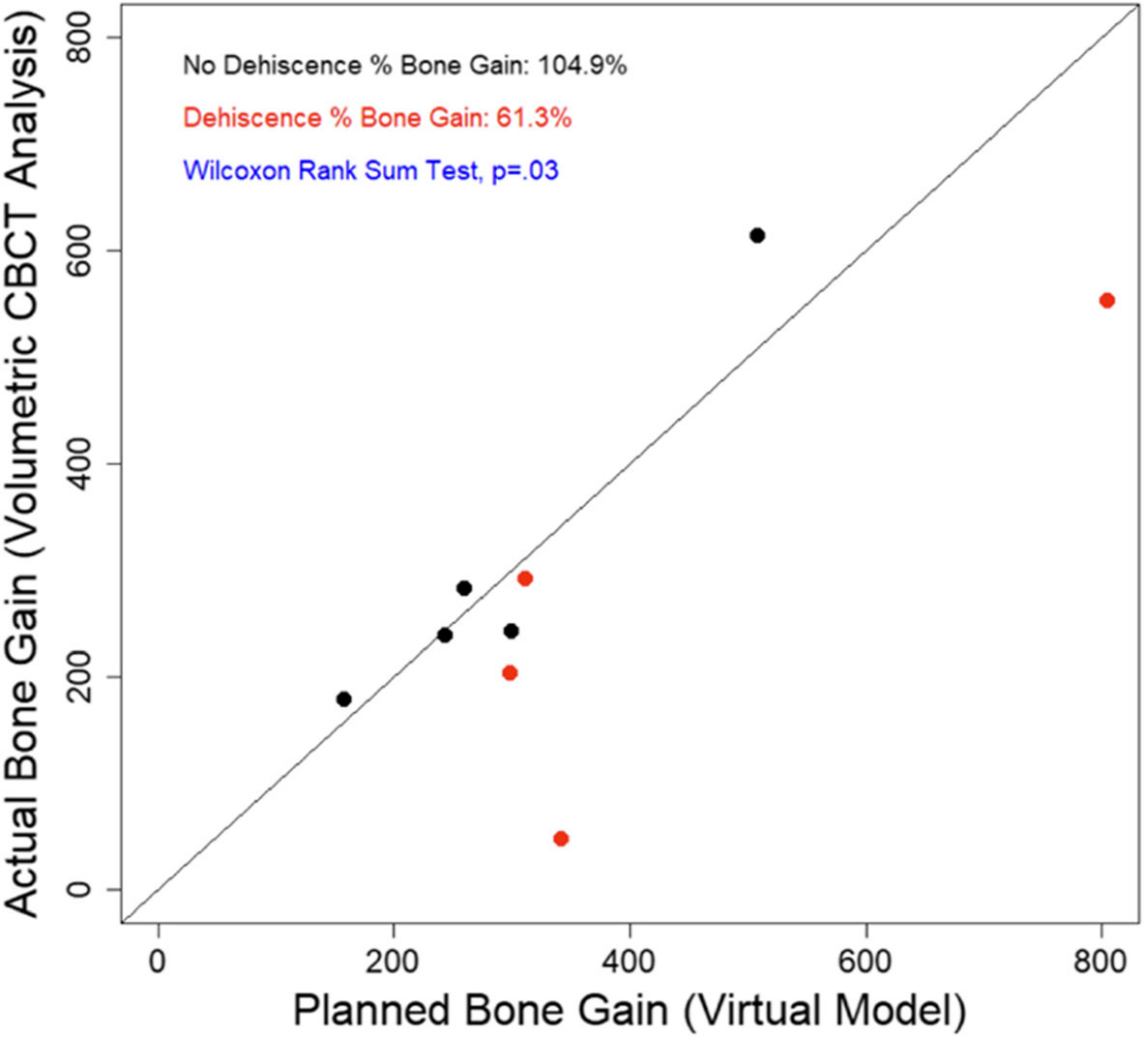
Planned vs. actual augmentation volume. Amount of volumetric bone gain (mm^3^) vs. the planned volume of augmentation (mm^3^). Red points represent subjects who experienced premature mesh exposure

The overall mean percentage bone fill as assessed through volumetric analysis was 85.5 ± 30.9%. Volumetric results are summarized in Table 1. In cases where premature mesh exposure did not occur, the mean percentage bone fill was 104.9 ± 15.5%. In the four cases where CTRAM became exposed prematurely, the mean percentage bone fill was 61.3 ± 33.7% (*p* = 0.03, Wilcoxon rank-sum test). Volumetric bone gain stratified by soft tissue outcome is displayed in Table 2 and Fig. 8.

**Table 1:** Volumetric bone gain

Subject ID	Baseline volume (mm^**3**^)	Virtual model increase (mm^**3**^)	Post-grafting volume (mm^**3**^)	Percentage bone fill
**1**^**a**^	0	311.29	292.06	93.82
**2**	0	507.44	614.64	121.13
**3**	0	259.70	283.34	109.10
**4**	0	242.68	238.84	98.42
**5**^**a**^	0	804.80	553.93	68.83
**6**^**a**^	0	297.45	203.88	68.54
**7**^**a**^	0	341.20	47.74	13.99
**8**	0	299.01	243.33	81.38
**9**	0	157.23	179.60	114.23

^a^Subjects with premature CTRAM exposure during healing

**Table 2:** Volumetric gain stratified by the occurrence of premature CTRAM exposure

Case outcome	Mean planned augmentation volume (mm^**3**^)	Mean post-graft volume (mm^**3**^)	Mean percentage bone fill
Premature CTRAM exposure	438.687	274.403	61.296 ± 33.69
No premature CTRAM exposure	293.211	311.948	104.851 ± 15.52
***p*** **value**^**a**^	**0.287**	**0.778**	**0.0355**

^a^Wilcoxon rank-sum test

### Assessment of CTRAM placement

The overall average discrepancy between planned CTRAM position and the actual position following surgical placement was 1.09 ± 0.81 mm (range 0.31–2.92 mm). These results are summarized in Table 3.

**Table 3:** CTRAM placement discrepancy

Subject ID	Point 1 difference (mm)	Point 2 difference (mm)	Point 3 difference (mm)	Point 4 difference (mm)	Mean difference (mm)
**1**	0.56	0.31	0.31	0.41	0.39
**2**	0.26	0.09	0.35	0.54	0.31
**3**	1.07	1.13	0.76	0.68	0.91
**4**	0.65	0.98	0.83	0.92	0.85
**5**	N/A	N/A	N/A	N/A	N/A
**6**	0.89	1.61	1.97	0.85	1.33
**7**	N/A	N/A	N/A	N/A	N/A
**8**	2.47	2.20	3.64	3.37	2.92
**9**	0.47	0.90	1.16	1.11	0.91
**Overall**					**1.089 ± 0.81**

Linear distance in millimeters from designated points on CTRAM: planned location (in CAD software) vs. location on post-treatment CBCT. Post-treatment CBCT on subjects 5 and 7 (early CTRAM removal) were obtained following removal of CTRAM, and positional comparison could not be made

Post-treatment CBCT scans on subjects 5 and 7 where CTRAM were removed early at the discretion of treating providers were not obtained with CTRAM still in place, and thus, placement discrepancy could not be assessed.

### Clinical evaluation of ridge augmentation

Clinical probing data, assessed at baseline and at re-entry, is summarized in Table 4. The mean baseline vertical probing measurement through the CTRAM was 4.49 ± 0.71 mm, and the mean baseline horizontal probing measurement was 3.91 ± 0.50 mm. The mean horizontal and vertical increases in ridge dimension achieved, assessed by comparing pre- and post-graft probing measurements, were 3.02 ± 0.84 mm and 2.86 ± 1.09 mm, respectively. Clinical measurements at the time of removal were not obtained on subjects #5 and 7, whose CTRAM were removed early.

**Table 4:** Clinical bone augmentation measurements

Subject ID	Mean baseline horizontal (mm)	Mean baseline vertical (mm)	Mean final horizontal (mm)	Mean final vertical (mm)	Mean horizontal gain (mm)	Mean vertical gain (mm)
**1**	3.875	5.2	1.75	2.5	2.125	2.7
**2**	4.125	5.6	0.5	1	3.625	4.6
**3**	4.5	5	0.125	1.25	4.375	3.75
**4**	4	3.8	0.25	0.625	3.75	3.175
**5**	4	4.2	–	–	–	–
**6**	4.5	5	1.25	2.5	3.25	2.5
**7**	3.5	4.4	–	–	–	–
**8**	4	3.8	1.75	3	2.25	0.8
**9**	2.75	3.4	0.625	0.5	2.125	2.9
**Overall**	**3.92 ± 0.50**	**4.49 ± 0.71**	**0.89 ± 0.63**	**1.63 ± 0.94**	**3.024 ± 0.84**	**2.86 ± 1.09**

Average of linear measurements through CTRAM at designated locations

## Discussion

Ridge augmentation using CTRAM is a viable modality for site development to facilitate placement of dental implants. As in previous studies that have outlined the advantages of a CAD/CAM methodology for rapid prototyping of titanium mesh [8, 9, 14], surgical providers participating in this study noted a relative ease of use and time savings compared to other modalities that require manual adaptation of a stock barrier material. The principal challenges with the CTRAM, as with any ridge augmentation technique, are careful soft tissue management and attainment of tension-free primary closure to minimize the risk of premature mesh exposure.

In this study, when CTRAM did become exposed prematurely, significantly less mean bone fill was achieved (61.3% vs. 104.9% when no exposure occurred). Machtei et al. [17] described the impact on the amount of bone regeneration achieved by the shift from an essentially sterile graft environment with complete soft tissue closure maintained to one that is contaminated by bacteria following wound dehiscence. In their review of relevant literature, they found that 24 non-exposed GBR sites gained 6 times as much new bone (3.01 ± 0.38 mm) compared with 36 sites (0.56 ± 0.45 mm) that became exposed.

A systematic review on alveolar bone augmentation with stock titanium mesh by Rasia dal Polo et al. reported an overall premature mesh exposure rate of 16.1%, and it was noted that in half of the studies reviewed early exposure led to a decrease in bone gain [18].

The rate of early CTRAM exposure in the present study (4/9 cases, 44.4%) is similar to findings reported in a recent pilot study by Ciocca et al. [9], who reported early exposure in 6/9 cases using similar methodology. Sagheb et al. observed a 33% rate of exposure in 21 sites augmented with customized titanium mesh [15]. Early mesh exposure does not seem to necessitate immediate removal of the CTRAM if measures such as twice-daily application of topical application of 0.12% chlorhexidine gluconate and regular post-operative monitoring are taken [9, 14].

The present study also found a much wider range (13.99–98.22%) in bone fill achieved in sites that experienced early mesh exposure versus sites that did not (81.38–121.13%). This suggests that another consequence of early exposure is less predictability with respect to the amount of bone regeneration that can be expected. Despite the diminished bone fill occurring with early mesh exposures, implant placement was nonetheless possible after augmentation in all cases in this study.

Several sites were found to have achieved over 100% bone fill, which can be explained by several factors. The reference volume, representing the denominator of the volume calculation, consists of the volume bound by the alveolar ridge at baseline and the CTRAM in its digitally planned location. When the CTRAM is fixated during surgery, slight deviations in placement versus the planned position can result in a larger potential augmentation volume. Additionally, the bone was shown in several cases to have regenerated beyond the dimension bound by the intaglio surface of the CTRAM, particularly in the region of the large bone loading ports. This is illustrated in Fig. 7c, where the pink surface represents the digitally planned augmentation, and the green represents the actual bone fill.

In all but one case, the mean deviation in CTRAM placement relative to the planned position was less than 1.5 mm. Even in the case with the highest mean deviation (subject 8, 2.92 mm), the deviation did not prevent dental implant placement in the correct position. The fact that this site was not tooth-bound (site #19 with teeth numbers 17 and 18 also missing) likely contributed to CTRAM becoming inadvertently fixated in a stable, but distally displaced, location. The degree of deviation is noteworthy in this case however, because the volumetric bone fill achieved (81.4%) was the lowest of all cases not experiencing premature mesh exposure.

## Conclusions

The use of CTRAM in conjunction with bone allograft and a collagen membrane for ridge augmentation resulted in vertical and horizontal bone gain suitable for implant placement in all nine subjects. Premature exposure of the mesh occurred in 4/9 subjects (44.4%), but this did not preclude successful implant placement.

## Data Availability

The datasets generated and analyzed during the current study are available from the corresponding author upon reasonable request.
